# Residues in the 1st Transmembrane-Spanning Helix Are Important for GABA_A_*ρ* Receptor Function

**DOI:** 10.3390/biom12091251

**Published:** 2022-09-07

**Authors:** Kate M. Crowther, Susanne M. Mesoy, Sarah C. R. Lummis

**Affiliations:** Department of Biochemistry, University of Cambridge, Tennis Court Road, Cambridge CB 1QW, UK

**Keywords:** Cys-loop receptor, binding site, mutagenesis, aromatic interaction, hydrophobic interaction

## Abstract

GABA_A_*ρ* receptors are a subfamily of the GABA_A_ receptor family of pentameric ligand-gated ion channels (pLGICs). Each subunit has a common structure, including a transmembrane domain of four α-helices (M1–M4). The aim of this study was to identify important M1 residues in the GABA_A_*ρ* receptor (GABA_A_*ρ*R), using mutagenesis and functional assays combined with bioinformatic approaches. Alanine substitution of 12 of the 23 M1 residues yielded receptors with altered functional parameters, indicating these residues contribute to GABA_A_*ρ*R function. Further mutations reveal the properties that are important for function in critical residues, and, using a GABA_A_*ρ*R homology model, we suggest amino acid interactions that could be important. Phylogenetic analysis comparing GABA_A_R and other pLGICs subunits reveals most M1 residue properties linked to GABA_A_*ρ*R function are ancestrally ancient, but some are more recent acquisitions. Multiple sequence alignment of M1 residues across GABA_A_R subunits reveal three residues are well conserved except in GABA_A_R α subunits. Substitution of *ρ*1 subunit residues to their α1 subunit equivalents showed one alters functional parameters. Overall, the data provide a comprehensive picture of M1 residues that contribute to GABA_A_*ρ*R function, and illustrate how they might do so.

## 1. Introduction

Pentameric ligand-gated ion channels (pLGICs), typified by the nicotinic acetylcholine (nACh) but also including GABA_A_, glycine, and 5-HT_3_ receptors, are primarily responsible for fast synaptic transmission in the central nervous systems of both vertebrates and invertebrates [1]. They are activated by the binding of neurotransmitters such as acetylcholine (ACh) or γ-aminobutyric acid (GABA), which induces a structural change in the protein causing the opening of an integral ion channel, and allowing ion flux across the post-synaptic membrane.

There are multiple isoforms of all pLGICs, but there is an especially large number for GABA_A_Rs, as different combinations of 19 possible subunits (α_1-6_, β_1-3_, γ_1-3_, δ, ε, π, θ, and/or *ρ*_1-3_) can contribute to each pentamer [2]. Different isoforms are expressed in different locations throughout the nervous system, with subunit composition influencing functional and pharmacological properties. GABA_A_*ρ*1Rs, for example, are mostly expressed in the retina, whereas GABAα1β3γ2R are broadly expressed throughout the CNS [3]. Functionally comparing these two receptors types reveals GABA is 10-100x more potent in GABA_A_*ρ*1R, channel opening times are slower, and the receptors are insensitive to neuromodulatory compounds such as barbiturates and benzodiazepines.

The receptors have a conserved basic structure, with each of the five subunits possessing a large N-terminal, extracellular domain (ECD), and a transmembrane domain (TMD) consisting of four membrane-spanning segments (M1-M4, of which M2 lines the ion pore). The ECD and TMD are covalently linked through pre-M1 residues, and non-covalently linked at the ECD-TMD interface through several conserved loops. Most pLGICs also contain an intracellular domain (ICD), which plays a role in ion conductance and receptor modulation. Neurotransmitter binding occurs between adjacent subunits in the ECD, and generates conformational changes that ultimately open the pore.

Previous studies have revealed which regions are involved in the mechanism of action of pLGICs, but the specific role of residues in many parts of pLGICs remains poorly understood. The cryo-EM revolution has recently made available the high-resolution structures of many GABA_A_R structures, enabling more detailed analysis of structure–function relationships [4,5,6,7]. These data, in combination with functional assays, have begun to provide evidence for how and why certain TMD residues contribute to receptor function, e.g., [8,9].

Nevertheless, many components of the TMD remain largely unstudied in GABA_A_Rs, and one such component is the M1 helix. This helix is both part of the outer ring of TMD helices (along with M3 and M4) that contact the lipid environment and neighbouring subunits, and is also situated near the pore-lining M2 helix and ECD structures such as the Cys loop. In other pLGICs, specific M1 helix residues have been shown to be important for receptor function, e.g., aromatic/hydrophobic M1 helix residues form interactions with the ECD, TMD helices in the same subunit, and/or TMD helices/loops in neighbouring subunits [10,11,12]. The aim of this study was to provide such information for the GABA_A_*ρ*1R, a typical member of the GABA_A_R family, using functional and bioinformatic studies. The data reveal that many residues in the M1 helix are important for GABA_A_*ρ*R function, and we propose this is probably through interactions with other key regions or components involved in the channel gating mechanism.

## 2. Materials and Methods

### 2.1. Molecular Biology

GABA_A_*ρ*1 (UniProt P24046) mutant receptor DNA was obtained by QuikChange site-directed mutagenesis (Agilent Technologies, Milton Keynes, UK) of the gene in pcDNA3.1 (Thermo Fischer Scientific, Paisley, UK), and the desired sequence was confirmed by nucleotide sequencing.

### 2.2. Cell Culture

Human embryonic kidney (HEK) 293 cells (ATCC, Teddington, UK) were maintained on 90 mm tissue culture plates at 37 °C and 7% CO_2_ in a humidified atmosphere. They were cultured in Dulbecco’s Modified Eagle’s Medium/Nutrient Mix F12 (DMEM: 1:1) with GlutaMAX I (Thermo Fischer Scientific) containing 10% HyClone fetal calf serum (GE Healthcare, Hatfield, UK). For FlexStation (Molecular Devices, Wokingham, UK) studies, cells were transfected using polyethylenimine (PEI; Merck, Gillingham, UK): 30 μL PEI (1 mg/mL), 5 μL cDNA (1 mg/mL; subcloned into pcDNA3.1), and 1 mL DMEM were incubated for 10 min at room temperature, added dropwise to a 70–90% confluent plate, and incubated for 2 days. Cells were then transferred to poly-l-lysine (Cultrex)-coated 96-well plates (Greiner BioOne, Stonehouse, UK) and allowed to adhere overnight before use.

### 2.3. Flexstation Studies

These methods were as described previously [13]. In brief, fluorescent membrane potential dye (Membrane Potential Blue kit, Molecular Devices) was diluted in Flex buffer (10 mM HEPES, 115 mM NaCl, 1 mM KCl, 1 mM CaCl_2_, 1 mM MgCl_2_, and 10 mM glucose, pH 7.4) and added to each well. The cells were incubated at 37 °C for 45 min and fluorescence measured in a FlexStation 3 (Molecular Devices) at 2 s intervals for 400 s. GABA (Merck) was added to each well after 20 s. Peak fluorescence (F) at each [GABA] was normalised to the maximum ΔF, and data were analysed using Prism (v6, GraphPad Software Inc., San Diego, CA, USA), fitting concentration–response data to the four-parameter logistic equation: $F=Fmin+\frac{\left(Fmax-Fmin\right)}{1+10^{(\log\left(EC_{50}\left[L\right]\right)n_{H}}}$, where [L] is the ligand concentration, n_H_ is the Hill coefficient, and Fmax and Fmin are the maximal and minimal fluorescence levels for each dataset (NB n_H_ values are reported but not discussed as it is difficult to meaningfully interpret these values when using an indirect assay). Statistical analysis was performed using ANOVA with a Dunnett’s multiple comparisons post test.

### 2.4. Multiple Sequence Alignment

The amino acid sequences for the 19 human GABA_A_R subunits (GABAα_1-6_, β_1-3_, γ_1-3_, δ, ε, θ, π, *ρ*_1-3_) and a representative sample of pLGIC subunits (GlyR α_1_, GluClR α, nAChR α_2_, 5-HT_3_A, ELIC, GLIC) were aligned using Clustal Omega (v1.2.4; EMBL-EBI, Cambridge, UK). UniProt accession numbers: P24046, P28476, A8MPY1, P14867, P47869, P34903, P48169, P31644, Q16445, P18505, P47870, P28472, Q8N1C3, P18507, Q99928, O14764, P78334, O00591, Q9UN88, P07727, Q94900, Q15822, P46098, P0C7B7, and Q7NDN8.

### 2.5. Generation of the GABA_A_ρR Homology Model and Structural Analysis

The human GABA_A_*ρ*1 and GABA_A_β3 subunit amino acid sequences (P24046 and P28472) were aligned using Clustal Omega. These sequences were used in Modeller (v9.25, MODELLER, San Francisco, CA, USA) alongside the GABA_A_β3 homopentamer crystal structure template (PDB 4COF) to generate a homology model for the GABA_A_*ρ*R. PyMOL (v2.4., Pymol, Cambridge, UK) was used to view structures and search for potential interactions by identifying residues within 5 Å of the target residue. Plausible cation–π interactions were determined using CAPture [14].

### 2.6. Phylogenetic Analysis

PhyML (v1; PHYML; Montpellier, France) was used to generate a phylogenetic tree from the multiple sequence alignment of all 19 GABA_A_R subunits and representative sample of pLGIC subunits using default parameters [15]. Ancestral sequences were predicted with FastML (v1; FastML, Tel Aviv, Israel) using default parameters [16].

## 3. Results

### 3.1. M1 Alanine Substitution

**Figure 1:**
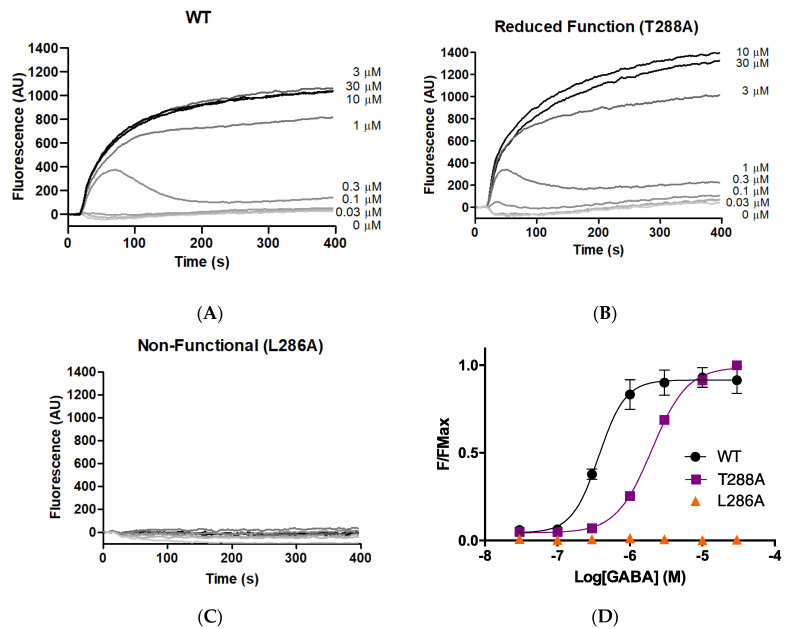
**Responses of GABA_A_***ρ***1R expressed in HEK293 cells.** (**A**–**C**) Typical fluorescent responses (fluorescence in arbitrary units, AU) on addition of GABA (0.03–30 μM) at 20 s to cells expressing WT or mutant GABA_A_*ρ*R. (**D**) Concentration–response curves; data = mean ± SEM, *n* ≥ 4.

As an initial probe to determine whether M1 helix residues make important contributions to GABA_A_*ρ*R function, each of the 23 GABA_A_*ρ*R M1 residues was systematically substituted to alanine. The WT and 23 alanine-substituted mutants were expressed in HEK293 cells, and stimulated with a range of concentrations of GABA. Typical responses are shown in Figure 1, with parameters obtained from these and similar data in Table 1. Parameters obtained for the WT GABA_A_*ρ*R were consistent with previously published data [8,9,17].

Alanine substitution of seven residues (F283A, F284A, L285A, L286A, Y289A, P291A, F303A) yielded receptors with little or no GABA-induced response, indicating these residues are important for some aspect of GABA_A_*ρ*R function (expression, folding, localisation, binding, and/or gating). Of the remainder, three of the substitutions (F282A, T288A, W304A) yielded receptors with a decrease in pEC_50_ compared to WT, indicating reduced receptor function, and two (M295A, M297A) had an increase, indicating a gain of function. Eleven of the 23 M1 alanine substitutions yielded receptors with WT-like responses.

### 3.2. Further M1 Substitutions

The alanine scan revealed that many M1 helix residues make important contributions to GABA_A_*ρ*R function, but did not reveal which residue properties are required. To test this, hydrophobic residues L285 and L286 were mutated to Ile and Val (hydrophobic but a different shape) and Asp (a similar shape but reduced hydrophobicity and negatively charged), while aromatic residues F283, F284, Y289, and F303 were mutated to Tyr/Phe and Trp (aromatic with similar shape/hydrophobicity), His (similar shape with reduced hydrophobicity, partial aromaticity, and partially positively charged), and Glu (different shape, reduced hydrophobicity/aromaticity, and negatively charged).

The data (Table 2) suggest hydrophobicity is a property required for L285 and L286 to contribute to GABA_A_*ρ*R function, as substitution to Asp (reduced hydrophobicity) yielded non-functional receptors. For L286 shape was also a required property, as even when substituted to Ile receptors remained non-functional. Aromaticity was a required property for contribution to GABA_A_*ρ*R function for F283, F284, Y289, and F303. Substitution to Glu (loss of aromaticity) yielded non-functional receptors and mutation to Phe/Tyr and Trp (maintained aromaticity) yielded functional receptors. For F283 and F284, substitution to His (a residue with partial aromatic nature) yielded receptors with some evidence of function, supporting our hypothesis that aromaticity is a required property.

For Y289 and F283, aromaticity was required, while for F284 and F303 shape was also important: substitution to Tyr (maintained aromaticity, similar shape, reduced hydrophobicity) yielded receptors with WT-like responses, but substitution to Trp (maintained aromaticity different shape, reduced hydrophobicity) yielded receptors with reduced function for F284 and no function for F303.

### 3.3. Phylogenetic Analysis

**Figure 2:**
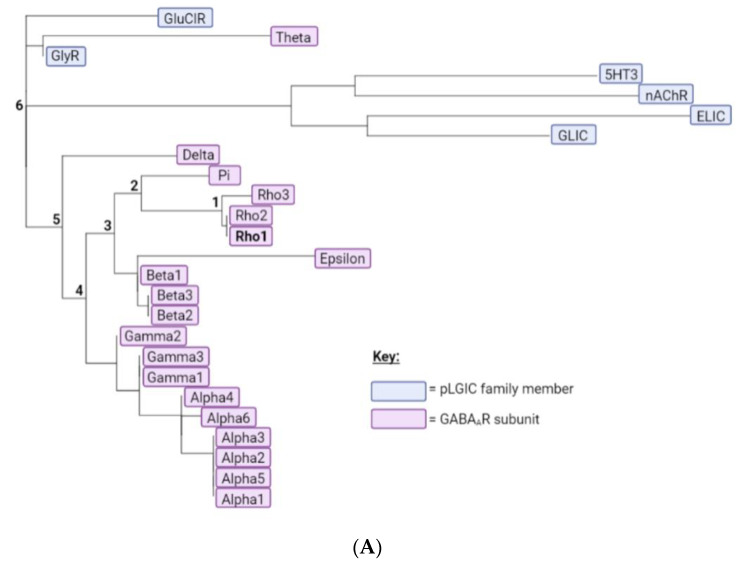

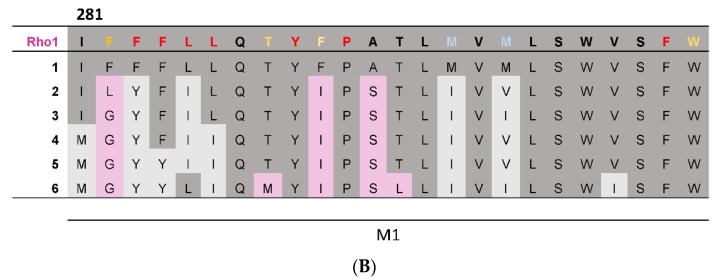
**A phylogenetic tree and predicted ancestral sequences.** (**A**) A phylogenetic tree of the 19 GABA_A_R subunits and representative subunits from other pLGIC family members, generated using the PhyML web server. GABA_A_R subunits are highlighted in pink and pLGIC family members in blue. Branches leading from the GABA_A_*ρ*1 subunit to the most ancestral sequence are labelled 1–6. (**B**) A multiple sequence alignment comparing the M1 helix of the GABA_A_*ρ*1 subunit to theoretical predicted ancestral sequences. If a residue is conserved between the GABA_A_*ρ*1 subunit and a given ancestral sequence, it is dark grey; if the general properties of the residue are conserved, residues are light grey; if not, residues are pink. Results from the M1 Ala substitution (Table 1) are also displayed: red = little or no function; orange = reduced function; blue = enhanced function; black = WT-like.

To provide complementary computational evidence, PhyML was used to construct a phylogenetic tree consisting of the 19 GABA_A_R subunits together with representative subunits from other pLGICs (Figure 2A). The predicted ancestral amino acid sequences were then calculated at the division of each branch using FastML, and the predicted M1 helix ancestral sequences assembled into a multiple sequence alignment (Figure 2B). This revealed most of the GABA_A_*ρ*1 subunit residues were present or had similar properties in the most ancestral sequence, indicating these may make similar contributions to function in all GABA_A_R. Nevertheless three residues were more recent acquisitions (F282, F290, A292), and thus may make GABA_A_*ρ*-specific contributions.

### 3.4. GABA_A_ρ1R to GABAα1 Substitution

**Figure 3:**
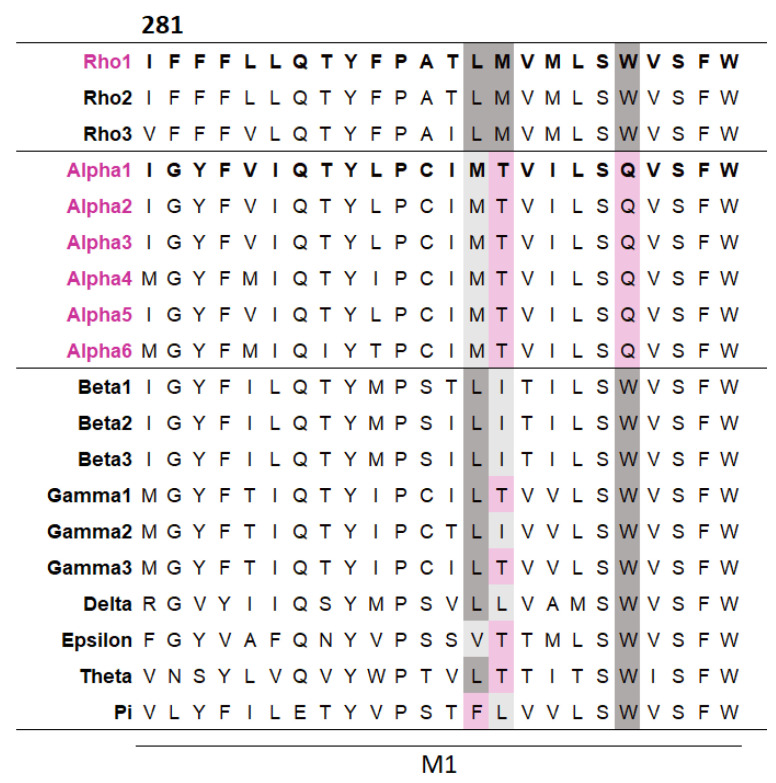
**A multiple sequence alignment reveals several residues in M1 differ in α subunits but are conserved across other GABA_A_R subunits.** The alignment was generated by Clustal Omega from the M1 regions of the 19 GABA_A_R subunits (the residue number shown is for the GABA_A_*ρ*1 subunit). Residues conserved between the GABA_A_*ρ*1 subunit and a given subunit are dark grey, residues with similar general properties to their GABA_A_*ρ*1 subunit equivalent are light grey, and non-conserved residues are pink.

To explore whether some TMD residues make subunit-specific contributions, a multiple sequence alignment of the M1 helices across the GABA_A_R subunits was generated. This revealed three residues that differed in GABAα subunits but were well conserved across other GABA_A_Rs (Figure 3). These different residues in the GABAα subunits may simply be the result of a harmless ancient mutation, with no selection pressure to be removed, or, more interestingly, they could make different contributions to function compared to other subunits. To investigate this, each of the three residues in the GABA_A_*ρ*1 subunit was substituted with their equivalent residue in the GABAα1 subunit and their function assessed (Table 3).

One of the three substitutions (M295T) yielded receptors with a significantly decreased pEC_50_ compared to WT, indicating the contribution of this residue is likely to be subunit specific. The remaining substitutions yielded receptors with WT-like responses; hence, we consider that these residues are unlikely to make major contributions to GABA_A_*ρ*R function.

## 4. Discussion

This study of the M1 helix reveals that many M1 residues make important contributions to GABA_A_*ρ*R function. The initial Ala scan showed that 12 of the 23 M1 residues are important to some aspect of GABA_A_*ρ*R function (i.e., expression, folding, localisation, binding, and/or gating), with 10 mutant receptors having reduced or no function, and two having enhanced function. Further substitution identified that hydrophobicity (I281, L285, L286, F284, F303), aromaticity (F283, F284, Y289, F303), and/or shape (F284, L286, F303) are properties required for contribution to GABA_A_*ρ*1R function. Phylogenetic analysis revealed that most properties of M1 residues are ancestrally ancient, and a multiple sequence alignment revealed three residues that differed in GABAα subunits but were well conserved in other subunits. Substituting each of these in GABA_A_*ρ*1R to their equivalent residue in the GABAα1 subunit revealed one has a subunit-specific contribution to GABA_A_*ρ*1R function. These findings, combined with the use of a GABAp1R homology model, and with previous evidence on the role of M1 residues in a range of pLGIC, enable hypotheses to be made of how M1 residues could contribute to function by interacting with nearby residues, structures, and/or lipids; these are discussed in more detail below.

**Figure 4:**
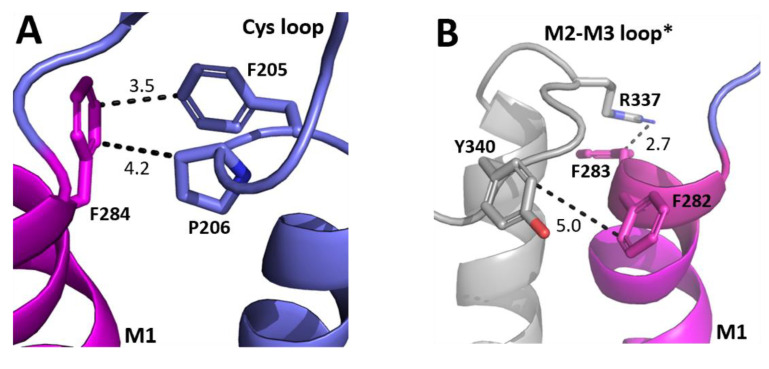
**Possible M1 helix residue interactions with the ECD.** (**A**) The interface between the top of the M1 helix (magenta) and the ECD Cys-loop (blue). (**B**) The interface between the top of the M1 helix (magenta) and the M2–M3 loop of the neighbouring subunit (grey). Distances are in Å. * indicates a neighbouring subunit.

A widely proposed critical interaction that links binding to pore opening is the interaction between the top of M1 and the Cys-loop. In the GABA*ρ*R, one residue that we identified as important, F284, is < 5 Å from the Cys loop residues F205 and P206 (Figure 4A). P206 contributes to a cis-peptide bond here, and this is facilitated by the adjacent F205 [18,19]. Nearby residues can help stabilise this bond and we propose that an F284–F205 π–π interaction and/or an F284-P206 proline–aromatic CH–π interaction is important for this, and thereby allows GABA_A_*ρ*R function. This hypothesis is consistent with the results from the non-Ala substitutions which revealed aromaticity was needed for the residue at position 284. Aromaticity here is conserved across equivalent residues in other GABA_A_R subunits and in most pLGICs (Figure 3), and mutation to Ala of this residue in GluCl (F276A) and GlyR (Y233A) yields non-functional receptors, while substitution to another aromatic does not [20,21]. Thus, these π–π and/or CH–π interactions with the Cys-loop Pro are likely conserved and critical for receptor function in the whole pLGIC family.

Another loop that interacts with M1 is the M2–M3 loop, and our model reveals that F282 and F283 are close to the M2–M3 loop of the neighbouring subunit (Figure 4B). F282 is ~5 Å from Y340, and thus might form a π–π interaction here, although the distance is not optimal and the Ala mutant still functions, so we suggest its role is to contribute to the local environment. In contrast aromaticity is essential at F283, and the model shows it could form a cation–π interaction with R337. Aromaticity is conserved here across most GABA_A_R subunits and in many other pLGICs (Figure 3), where it can also affect function, e.g., in the GlyR the equivalent Y222 yields non-functional receptors when substituted with Ala but not with an aromatic residue [10,11,12].

Farther down M1, L285 and L286 face outwards, and we suggest they contribute to GABA_A_*ρ*R function by forming interactions with lipids in the membrane. Lipid interactions are increasingly being appreciated as important for pLGIC function, with some receptors tightly bound to specific lipids (e.g., PIP2 to the GABA_A_R [5]). Such interactions may be purely hydrophobic, and our data suggest this is the case for L285, but shape is also important in some locations, and this may be why L286 is sensitive to replacement even with residues with similar properties. This Leu is conserved across GABA_A_R subunits (Figure 3) but not in other pLGICs, although a hydrophobic residue is usually present. The importance of these residues to function in other pLGICs varies: GlyRs with L224A or I225A substitutions yield receptors with WT-like responses, L204A in ELIC yields receptors with reduced function, and I198A in GLIC yields non-functional receptors [10,11]. Such differences are consistent with different lipid interactions with differing importance in different pLGICs.

**Figure 5:**
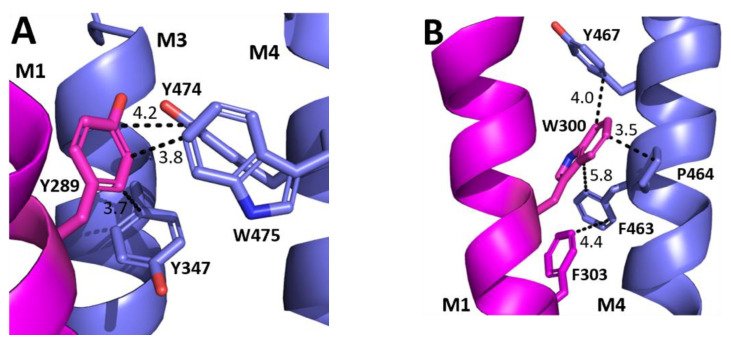
**Possible M1 helix interactions with M3 and M4 helices.** (**A**) The interface between the M1 (magenta) and M3 and M4 helices (blue) close to Y289. (**B**) The interface between the M1 (magenta) and the M4 helix (blue) close to W300 and F303.

**Figure 6:**
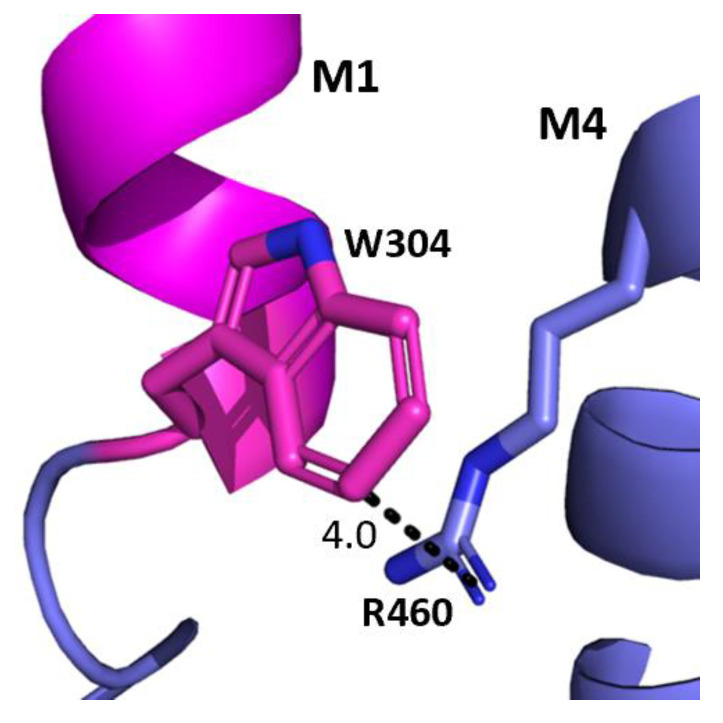
**Potential W304-M4 interaction.** The interface between the M1 helix (magenta) and the M4 helix (blue) showing the potential cation–π interaction between W304 and R460. Distances are in Å.

The aromatic residues situated toward the intracellular side of M1 could contribute to communication between transmembrane helices. Y289 and F303 are situated within hydrophobic clusters composed of M1, M3, and/or M4 residues, and could make π–π and CH–π interactions with these residues (Figure 5). Our data showed that aromaticity is required here and indeed aromaticity is conserved across GABA_A_R subunits as well as in most other pLGICs, where it is important, e.g., in the GlyR Y228A (the residue equivalent to Y289) yields non-functional GlyR receptors and F242A/F216A (F303 equivalent residues) yield GlyR or GLIC receptors with reduced function [10,11,12]. Other aromatic residues in the clusters have also been shown to contribute to GABA_A_*ρ*R function, e.g., Y474A and W475A substitutions yield receptors with reduced or no function, and F463A substitution yields non-functional receptors [8]. W304 is predicted to form a cation–π interaction with the M4 residues R460 (Figure 6), and thus could assist in transmitting information between these two helices. A Trp here is conserved in most pLGICs, with Ala substitution yielding non-functional receptors or receptors with reduced function in all pLGICs investigated [10,11,12], supporting an important role for this residue.

The final Ala M1 mutant that ablated function is P291. The structural importance of the M1 kink caused by Pro has been examined in both this and other receptors and will not be further discussed here [9,22,23].

**Figure 7:**
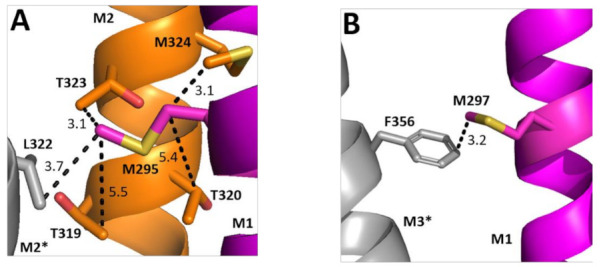
**Possible M1 helix Met interactions.** Residues close to M295 (**A**) and M297 (**B**) showing the M1 helix (magenta), the M2 helix (orange), and the neighbouring M3 helix (grey). Distances are in Å. * indicates a neighbouring subunit.

Two of the Ala substitutions cause a decrease in EC_50_. Examining their structural location reveals the hydrophobic M295 is in a relatively hydrophilic environment, although could interact with Leu on the adjacent M2 helix (Figure 7A). Replacement with Ala would remove this hydrophobic interaction and thus might favour conformational change. Conversely, M297 faces away from M2 and toward M3, with which it could form a Met–π interaction; we speculate that this rigid bond is unfavourable to conformational change and thus there is a gain of function when it is replaced by Ala.

## 5. Conclusions

In conclusion, we found that many M1 helix residues make contributions to GABA_A_*ρ*R function, and propose this is largely because they allow communication with other parts of the receptor. We await further structural data to test this hypothesis.

## Figures and Tables

**Table 1:** Parameters from concentration–response curves after Ala substitution of M1 helix residues.

Mutant	pEC_50_ (M)	EC_50_ (μM)	nH	n
WT	6.44 ± 0.03	0.35	2.2 ± 0.4	14
I281A	6.24 ± 0.11	0.58	1.3 ± 0.4	4
F282A	5.27 ± 0.06 *	5.60	1.4 ± 0.3	4
F283A	NF			6
F284A	NF			6
L285A	NF			6
L286A	NF			6
Q287A	6.33 ± 0.02	0.46	1.2 ± 0.1	4
T288A	5.72 ± 0.03 *	2.00	1.7 ± 0.1	4
Y289A	SR			6
F290A	6.90 ± 0.02	0.12	1.6 ± 0.1	4
P291A	SR			9
T293A	7.06 ± 0.02	0.09	1.4 ± 0.1	4
L294A	6.94 ± 0.06	0.11	3.5 ± 1.3	4
M295A	7.25 ± 0.05 *	0.06	2.4 ± 0.4	4
V296A	6.88 ± 0.06	0.13	1.5 ± 0.3	4
M297A	7.26 ± 0.08 *	0.06	1.1 ± 0.2	4
L298A	6.14 ± 0.09	0.71	1.8 ± 0.6	4
S299A	5.95 ± 0.01	1.10	2.5 ± 0.1	4
W300A	6.20 ± 0.05	0.64	1.2 ± 0.1	4
V301A	5.87 ± 0.04	1.39	1.8 ± 0.3	4
S302A	6.21 ± 0.13	0.61	0.7 ± 0.1	4
F303A	NF			6
W304A	5.72 ± 0.04*	1.90	1.1 ± 0.1	4

Data are mean ± SEM. NF = non-functional at up to 30 μM GABA. SR = small responses, so parameters could not be accurately calculated. * = significantly different from WT, *p* < 0.01, ANOVA with Dunnett’s multiple comparison test, and >5-fold change in EC_50_ compared to WT.

**Table 2:** Parameters derived from concentration–response curves after substitution of a selection of M1 helix residues.

Mutant	pEC_50_ (M)	EC_50_ (μM)	nH	n
WT	6.44 ± 0.03	0.35	2.2 ± 0.4	14
F283Y	6.06 ± 0.02	0.87	1.9 ± 0.2	4
F283W	6.83 ± 0.05	0.14	1.1 ± 0.2	4
F283H	SR			6
F283E	NF			6
F284Y	7.00 ± 0.40	0.09	1.8 ± 1.1	4
F284W	5.28 ± 0.03*	5.24	2.0 ± 0.2	4
F284H	SR			6
F284E	NF			6
L285I	6.73 ± 0.13	0.19	0.9 ± 0.2	4
L285V	6.81 ± 0.08	0.14	1.3 ± 0.2	4
L285D	NF			4
L286I	NF			6
L286V	NF			6
L286D	NF			6
Y289F	6.76 ± 0.04	0.17	3.1 ± 0.5	4
Y289W	6.73 ± 0.15	0.19	0.9 ± 0.3	4
Y289H	NF			6
Y289E	NF			6
F303Y	7.13 ± 0.05	0.08	2.0 ± 0.4	4
F303W	NF			6
F303H	NF			6
F303E	NF			6

Data are mean ± SEM. NF = non-functional up to 30 μM GABA. SR = small responses, so parameters could not be accurately calculated. * = significantly different to WT, *p* < 0.01, ANOVA followed by Dunnett’s multiple comparison test, and >5-fold change in EC_50_ compared to WT.

**Table 3:** Parameters derived from concentration–response curves of GABA_A_*ρ*1R after substitution with GABA_A_R α subunit M1 residues.

Mutant	pEC_50_ (M)	EC_50_ (μM)	nH	n
WT	6.45 ± 0.03	0.35	2.3 ± 0.3	12
L294M	6.51 ± 0.04	0.31	1.3 ± 0.1	4
M295T	5.76 ± 0.06 *	1.80	1.7 ± 0.4	4
W300Q	6.95 ± 0.13	0.12	1.1 ± 0.4	6

Data are mean ± SEM. * = significantly different values from WT, *p* < 0.01, ANOVA followed by Dunnett’s multiple comparison test, and > 5-fold change compared to WT.

## Data Availability

Data are available on request from the authors.
